# Phosphorylated Trehalose Suppresses the Denaturation of Myofibrillar Proteins in Peeled Shrimp (*Litopenaeus vannamei*) during Long-Term Frozen Storage

**DOI:** 10.3390/foods11203189

**Published:** 2022-10-13

**Authors:** Shanshan Shui, Huicheng Yang, Baiyi Lu, Bin Zhang

**Affiliations:** 1 Department of Food Science and Nutrition, College of Biosystems Engineering and Food Science, Zhejiang University, Hangzhou 310058, China; 2 Key Laboratory of Health Risk Factors for Seafood of Zhejiang Province, College of Food Science and Pharmacy, Zhejiang Ocean University, Zhoushan 316022, China; 3 Zhejiang Marine Development Research Institute, Zhoushan 316022, China

**Keywords:** phosphorylated trehalose, shrimp, myofibrillar proteins, frozen storage, denaturation, oxidation

## Abstract

The protective effects of phosphorylated trehalose on the quality and characteristics of peeled shrimp (*Litopenaeus vannamei*) were determined. Quality changes in treated samples were evaluated by assessing the physicochemical properties of myofibrillar proteins (MP) and compared to fresh water-, sodium tripolyphosphate-, and trehalose-treated samples during 12 weeks of frozen storage. The sensitivity of MP to oxidation and denaturation was increased during frozen storage. Phosphorylated trehalose significantly improved the quality of shrimp by increasing water-holding capacity. Further analysis showed that the addition of phosphorylated trehalose reduced the decrease in soluble MP content, Ca^2+^-ATPase activity, and total sulfhydryl contents and also effectively inhibited the increase in the surface hydrophobicity of MP. In addition, atomic force microscopy and hematoxylin and eosin staining revealed that phosphorylated trehalose preserved the integrity of the myofibril microstructure. Thermal stability results further confirmed that the denaturation temperature and denaturation enthalpy of MP were improved by phosphorylated trehalose. Overall, phosphorylated trehalose suppresses the denaturation of MP in peeled shrimp during long-term frozen storage.

## 1. Introduction

The Pacific white shrimp (*Litopenaeus vannamei*) are popular worldwide because of their delicious taste and good nutritional value. The production volume of Pacific whiteleg shrimp was approximately 5 million tons in 2018 reported by the Food and Agriculture Organization (FAO). Frozen storage is most commonly used to process, transport and preserve shrimp products, as it inhibits microbial growth, reduces enzyme activity and helps maintain shrimp flavor. However, some quality deteriorations, such as lipid oxidation, protein denaturation, and ice crystal formation and growth, still occur during long-term frozen storage, which negatively impacts consumer acceptance of shrimp products [1].

Phosphate (tripolyphosphate or a blend of different chain length phosphates) is widely used as a food-grade additive in frozen shrimp, which can promote water-holding capacity (WHC) and maintain the texture of shrimp [2]. However, excessive addition of phosphate causes the corresponding side effects for shrimp, such as metallic astringency, the roughness of muscle tissue and ice crystal formation of the frozen surface. Since shrimp contains phosphorus, long-term intake of shrimp products treated with phosphate may result in excessive phosphorus intake [3]. In addition, many countries have strict regulations on the maximum usage of phosphate levels in shrimp products (irrespective of source), and alternative additives with water retention should be considered.

Trehalose is a nonreducing disaccharide formed by condensing two glucose molecules through a hydroxyl group of hemiacetal and connected by a 1,1-glycosidic bond [4]. Trehalose exhibits potential cryoprotective effects on the stabilization of membranes and protein structure and inhibition of mechanical damage and protein denaturation in frozen shrimp [5]. Moreover, phosphorylation of saccharides can change the structural and conformational properties of saccharides, thus enhancing their physicochemical and biological activities [6]. Reports exist of sodium tripolyphosphate and sodium trimetaphosphate being used to modify polysaccharides from Trichosanthes peel [7], Amana edulisb [8] and birchwood xylan [9]. However, the study on the bioactivities of phosphorylated trehalose and its effects on frozen shrimp have not been reported. 

In this study, the effects of fresh water, sodium tripolyphosphate, trehalose and phosphorylated trehalose on the WHC, structure and functions of myofibrillar proteins (MP) of frozen Pacific white shrimp were investigated. Further, the cryoprotective mechanism of phosphorylated trehalose involved in protein denaturation of shrimp during frozen storage was elucidated. 

## 2. Materials and Methods

### 2.1. Chemicals

Trehalose (C_12_H_22_O_11_) and sodium tripolyphosphate (Na_5_P_3_O_10_) were obtained from Sinopharm Chemical Reagent Co., Ltd. (Shanghai, China). All other reagents were of analytical grade from Kemeo Reagent Co., Ltd. (Tianjin, China).

### 2.2. Preparation of Phosphorylated Trehalose

Trehalose was added to 9% (*w/v*) sodium tripolyphosphate aqueous solution to achieve a final concentration of 4% (*w/v*) trehalose, and 3.7% (*w/v*) NaHCO_3_ was used to adjust the pH to 8.0. The mixture was reacted for 7 h at 90 °C in a water bath with an oscillating sample holder. After cooling to room temperature, the reaction solution was desalted using D301 macroporous weak-base anion exchange resin and then precipitated with the six-fold volume of anhydrous ethanol for 24 h at 4 °C. Subsequently, the residue was recovered by centrifuging at 5000 g for 10 min. Finally, the residue was freeze dried to obtain phosphorylated trehalose powder. The radical phosphate content of phosphorylated trehalose was 12.29% (*w/w*) using extraction-phosphomolybdate blue spectrophotometry [10], and the weight-average molecular weight was 597.7.

### 2.3. Shrimp Sample and Treatments

Shrimp samples (*Litopenaeus vannamei*) with 20.0–22.5 g in weight and 9.5–11.5 cm in length were obtained from the Laoqi market in Zhoushan, China. The live shrimp were transported to the laboratory within 20–30 min after packaging in an incubator with ice cubes. On arrival at the laboratory, the shrimp were placed in a refrigerated box held at 4 °C, washed, decapitated and sorted by size. The shrimp were deveined after the cryopreservative soaking treatment. The K-value of samples was measured by RP-HPLC and found to be 2.0–2.5%. 

The peeled shrimp were randomly divided into four cryopreservative groups and soaked in the following solution at 4 °C: fresh water, 3.0% (*w/v*) sodium tripolyphosphate, 3.0% (*w/v*) trehalose, and 3.0% (*w/v*) phosphorylated trehalose. The concentration was designed based on our previous studies [11]. After soaking for 1 h, the shrimp were transferred from solution and drained for 1 min, followed by frozen at −30 °C for 3 h. Finally, the frozen shrimp from the different groups were packaged with polyethylene bags (20.0 cm × 25.0 cm, 150 μm thickness) and stored at −18 °C for 12 weeks, respectively. Subsequent analysis of triplicate samples (*n* = 3) from each cryopreservative group was performed every 3 weeks during frozen storage (0, 3, 6, 9, 12 weeks).

### 2.4. Determination of Water-Holding Capacity (WHC)

The thawing loss (%) of shrimp was determined by weighing the samples before and after thawing. 

The shrimp were steamed for 5 min so they reached an internal temperature of 95 °C. The cooking loss (%) of the frozen sample was measured by weighing the shrimp before and after cooking.

### 2.5. Extraction of Myofibrillar Proteins (MP)

All operations were performed at 4 °C. Minced shrimp (5.0 g) was added to 15 mL extraction buffer (20 mmol/L tris-maleate containing 0.05 mol/L KCl, pH 7.0) and homogenized for 60 s. The mixture was centrifuged at 10,000 g for 10 min, and the supernatant was discarded. The residue was re-suspended in extraction buffer, and the extraction was repeated once. At the end of the second centrifugation, the obtained precipitate was added with extraction buffer and centrifuged at 6000 g for 15 min. Finally, the supernatant was regarded as soluble MP, and its concentration was measured according to Zhang’s method [1].

### 2.6. Determination of Ca^2+^-ATPase Activity

The extracted MP (1.0–2.0 mg/mL) was added to reaction buffer (0.50 mol/L tris-maleate containing 20 mol/L ATP and 0.10 mol/L CaCl_2_, pH 7.0) and bathed at 30 °C for 5 min. 1.0 mL of 15% (*w/v*) TCA was added to the mixture and then centrifuged at 4000 g for 5 min. The Ca^2+^-ATPase activity of MP was assayed according to the amount of inorganic phosphate (Pi) released in the supernatant [12]. 

### 2.7. Determination of Total Sulfhydryl (T-SH) Content

MP was dissolved in 20 mmol/L tris-HCl buffer (pH 7.0) and then added to 50 mmol/L phosphate buffer. After centrifugation of the mixture, the supernatant was incubated with 10 mmol/L DTNB solution at 37 °C for 15 min. The T-SH content was determined based on the absorbance at 412 nm as the μmol of sulfhydryl groups per milligram of MP.

### 2.8. Determination of Surface Hydrophobicity

MP was dissolved in 20 mmol/L phosphate buffer (containing 0.6 mol/L NaCl, pH 7.0) and adjusted to a concentration gradient. Then, 0.1 mol/L phosphate buffer (containing 8 mmol/L 1-anilinonaphthalene-8-sulfonic acid (ANS), pH 7.0) was added to each protein solution. The relative fluorescence intensity of the sample was measured by a spectrofluorometer (F-4500 model, Hitachi, Tokyo, Japan) at 374 nm (excitation wavelength) and 485 nm (emission wavelength). The surface hydrophobicity of MP was calculated using linear regression of the initial slope (SoANS) of a plot of the relative fluorescence intensity to protein concentration. 

### 2.9. Atomic Force Microscope (AFM) Analysis

MP suspension was evenly spread on a newly cleaved mica and allowed to dry under ambient conditions. The AFM of MP was determined and photographed using a Park AFM (NX10, Park Systems Corporate, Wanggyo, Korea).

### 2.10. Hematoxylin and Eosin (H&E) Staining Analysis 

The shrimp were immersed in formalin fixative that contained ethanol, glacial acetic acid, and distilled water at room temperature for 15 h. The fixed samples were washed with 50% ethanol, dehydrated with 70%, 80%, 90%, 95%, and 100% ethanol, and further embedded in paraffin blocks. Next, the paraffin-embedded samples were cut into 5 µm-thick sections and stained with H&E. The sections were examined under light microscopy (BX51, Olympus Co., Ltd., Beijing, China).

### 2.11. Differential Scanning Calorimetry (DSC) Analysis

DSC was performed by a differential scanning calorimeter (model 200 F3, NETZSCH Group, Deutschland, Germany). A 15 mg of shrimp muscle tissue was sealed in a standard aluminum pan. Then, the sample pan and control pan (empty pan) were heated from 25 °C to 95 °C at 5 °C/min in the presence of nitrogen. The denaturation temperature (*Tmax*, °C) and enthalpy (Δ*H*, J/g) were used to characterize protein denaturation (primarily myosin and actin).

### 2.12. Data Analysis

Each result was subjected to statistical analysis in SPSS package 13.0 for Windows (SPSS Inc., Chicago, IL, USA). Two-way ANOVA and Duncan’s test were used to compare the group, and *p* < 0.05 was considered as a significant difference in the means.

## 3. Results and Discussion

### 3.1. Water-Holding Capacity (WHC) Analysis

WHC refers to the ability of the muscle to keep moisture during cutting, heating, grinding, extrusion, transportation and frozen storage. WHC is closely related to the degree of protein denaturation and proteolysis in shrimp muscle and is an important indicator of sensory quality and is economically important as many foods are sold based on their weight. 

The effects of fresh water, sodium tripolyphosphate, trehalose, and phosphorylated trehalose on the WHC of shrimp muscle during frozen storage are shown in Table 1. Thawing and cooking loss of all treated samples significantly increased with increased storage time, which was in accordance with the findings of previous studies [13]. The results may be due to physical damage to shrimp muscle tissue caused by the growth and recrystallization of ice crystals, leading to MP denaturation, the myofilaments lattice disintegration, including the I-band, H-band and Z-disc, and an increase in extracellular space [14]. 

Compared with fresh water-treated shrimp, sodium tripolyphosphate, trehalose, and phosphorylated trehalose treatments significantly increased the WHC, as indicated by the lower thawing and cooking losses. In addition, the effect of phosphorylated trehalose was notably better than those of sodium tripolyphosphate and trehalose. The two-way ANOVA (Tables S1 and S2) indicated that the WHC results were significantly influenced by the cryopreservative treatment, storage time and their interaction. Phosphates can increase sample pH, reduce the degree of freezing denaturation of MP, and increase the exposure probability of polar groups of protein molecules by chelating calcium, magnesium and other metal ions, thus enhancing the WHC of shrimp [3]. As a kind of polyhydroxy substance, trehalose can maintain the stability of protein structure by forming hydrogen bonds with the polar residues of the protein and then plays the role of water retention [11]. A possible explanation for the best WHC of phosphorylated trehalose-treated shrimp is the combination of the effective grafting of phosphate and cryoprotective properties of trehalose.

### 3.2. Myofibrillar Protein (MP) Content and Ca^2+^-ATPase Activity Analysis

**Figure 1:**
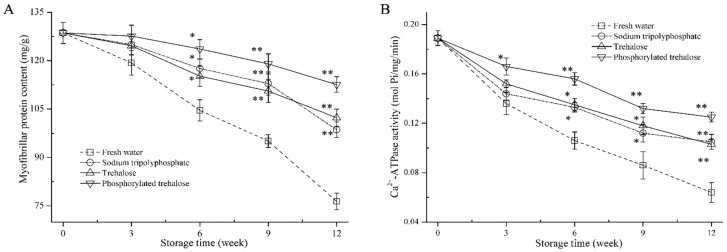
Myofibrillar protein content (**A**) and Ca^2+^-ATPase activity (**B**) in peeled shrimp soaked with fresh water, sodium tripolyphosphate, trehalose, and phosphorylated trehalose during frozen storage. * *p* < 0.05 and ** *p* < 0.01 compared with the fresh water treatment at the same point.

MP is the main component of shrimp muscle protein. The MP content and structure changes directly affect the functional characteristics of the protein. Myofibrillar Ca^2+^-ATPase activity is commonly used to measure MP integrity and denaturation. The soluble MP content and Ca^2+^-ATPase activity in peeled shrimp immersed in fresh water, sodium tripolyphosphate, trehalose, and phosphorylated trehalose were measured during 12 weeks of frozen storage, respectively (Figure 1). 

The two indexes of all batches in peeled shrimp muscle decreased significantly with frozen storage time. During the storage period, the highest rates of decrease in soluble MP content and Ca^2+^-ATPase activity were observed in fresh water-treated shrimp muscle. The soluble MP content and Ca^2+^-ATPase activity maintained in sodium tripolyphosphate, trehalose, and phosphorylated trehalose treatment were higher than those in fresh water samples. Notably, the effects of phosphorylated trehalose on soluble MP content and Ca^2+^-ATPase activity were greater than those of the other treatments after 12 weeks of frozen storage. The two-way ANOVA (Tables S1, S3 and S4) indicated that the soluble MP content and Ca^2+^-ATPase activity were significantly influenced by the cryopreservative treatment, storage time and their interaction. 

Water in shrimp muscle migrates to the extracellular spaces during freezing. It causes the increase in intracellular ionic strength, followed by the degradation of myofibrils and connective tissue in the muscle, leading to a decrease in MP content [15]. The myosin globular head may denature by the mechanical squeezing of ice crystals and increasing solutes concentration in the remaining liquid fraction during frozen storage, decreasing Ca^2+^-ATPase activity [16]. Commonly used phosphates are weakly alkaline, and thus can increase the sample pH, reduce the degree of freezing denaturation of MP, and improve protein stabilization [3]. The hydrophilic hydroxyls in trehalose combine water molecules and then lessen the formation and growth of ice crystals in the shrimp, avoiding changes to the MP conformation [17]. Phosphorylated trehalose effectively reduced the decrease in MP solubility and Ca^2+^-ATPase activity, which was likely associated with the enhancement of trehalose bioactivity through the extension of chain conformation in trehalose by phosphate groups [18].

### 3.3. Total Sulfhydryl (T-SH) Content and Surface Hydrophobicity Analysis

Sulfhydryl groups are the most reactive functional group on the myosin head portion and are easily oxidized to disulfide groups during freeze-thawing or frozen storage. The decrease in T-SH content of frozen shrimp muscle is commonly used to evaluate the degree of protein oxidation. 

**Figure 2:**
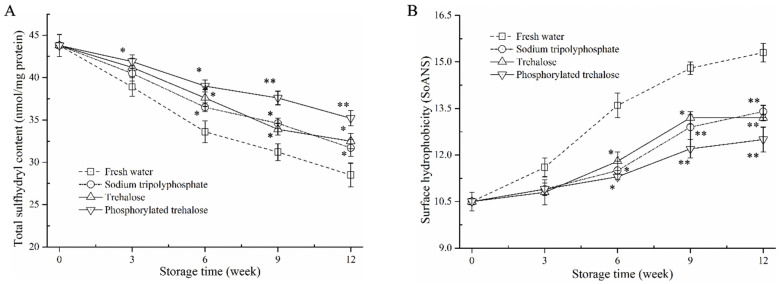
Total sulfhydryl content (**A**) and surface hydrophobicity (**B**) of MP in peeled shrimp soaked with fresh water, sodium tripolyphosphate, trehalose, and phosphorylated trehalose during frozen storage. * *p* < 0.05 and ** *p* < 0.01 compared with the fresh water treatment at the same point.

In the current study, the T-SH content of MP in shrimp muscle decreased observably with frozen storage time in all batches (Figure 2A). The initial T-SH content of the fresh sample was 43.8 nmol/mg protein. After 12 weeks of frozen storage, the T-SH content of fresh water-treated samples was reduced to 28.5 nmol/mg protein, suggesting the occurrence of protein oxidation in shrimp muscle [19]. However, samples treated with sodium tripolyphosphate (31.7 nmol/mg protein), trehalose (32.5 nmol/mg protein), and phosphorylated trehalose (35.2 nmol/mg protein) had higher T-SH levels than the fresh water-treated samples. The T-SH levels in the three treatments were similar during the initial 0–6 weeks of frozen storage. The two-way ANOVA (Tables S1 and S5) indicated that the T-SH content was significantly influenced by the cryopreservative treatment, storage time and their interaction. 

The higher T-SH contents may be related to the antioxidant activity of trehalose that can counteract oxidative stress by scavenging free radicals produced during frozen storage. Moreover, trehalose can improve the structural and functional stability of MP by forming hydrogen bonds with polar amino acids [20]. The phosphorylated trehalose treatment had higher T-SH values than the sodium tripolyphosphate and trehalose when the storage period was 9–12 weeks, indicating the stronger antioxidant activity of phosphorylated trehalose. In addition, the above results of Ca^2+^-ATPase activity also confirmed the positive correlation between sulfhydryl oxidation and Ca^2+^-ATPase activity [21].

Surface hydrophobicity is connected with the structural stabilization of MP and is generally performed to assess conformational changes in protein structure. The changes in the surface hydrophobicity of MP soaked in fresh water, sodium tripolyphosphate, trehalose, and phosphorylated trehalose during 12 weeks of frozen storage were shown in Figure 2B. 

The initial SoANS value in a fresh sample (0 week) was 10.5 MP due to slight binding of fluorescent probes to exposed hydrophobic groups. The fresh water sample value increased markedly to 15.3 after 12 weeks of storage. Similar increases were also found in shrimp samples treated with sodium tripolyphosphate, trehalose, and phosphorylated trehalose. Protein oxidation during frozen storage causes loose and destabilized conformational structures of MP, leading to the exposure of hydrophobic groups, which increase surface hydrophobicity [22]. The SoANS values in these three treatments increased to 13.4, 13.2, and 12.5, respectively, which were significantly less than the control value at the storage end. The inhibition of the upregulation of surface hydrophobicity was not significantly different among the sodium tripolyphosphate, trehalose and phosphorylated trehalose treatment. The two-way ANOVA (Tables S1 and S6) indicated that the surface hydrophobicity was only significantly influenced by the cryopreservative treatment.

As reported, some saccharides can replace water molecules and help stabilize the three-dimensional structure of the protein by forming hydrogen bonds with the hydrophilic residues on the protein surface [23]. The results demonstrated that the phosphorylated trehalose restrained the denaturation and aggregation progress of MP, predominantly due to its better antioxidant activities after phosphorylation, including free radical scavenging and metal ion chelating, and the hydrophilic–hydrophilic interactions between saccharides and MP [18,24]. 

### 3.4. Atomic Force Microscope (AFM) Analysis

**Figure 3:**
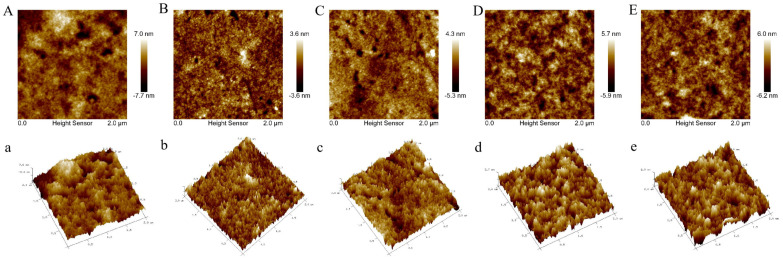
The 2D and 3D AFM images of MP in peeled shrimp soaked with fresh water, sodium tripolyphosphate, trehalose, and phosphorylated trehalose after 12 weeks of frozen storage. (**A**,**a**): fresh shrimp muscle tissue (0 d); (**B**,**b**): shrimp muscle tissue treated with fresh water; (**C**,**c**): shrimp muscle tissue treated with sodium tripolyphosphate; (**D**,**d**): shrimp muscle tissue treated with trehalose; (**E**,**e**): shrimp muscle tissue treated with phosphorylated trehalose.

The nanostructural changes of MP extracted from peeled shrimp after different treatments were determined using AFM (Figure 3). The light part denoted the convex areas of MP, and the dark part denoted the concave regions of MP. Significant differences were observed among samples soaked in fresh water, sodium tripolyphosphate, trehalose, and phosphorylated trehalose after 12 weeks of frozen storage. 

The images (Figure 3A,a) showed an intact, large nanostructure (altitude ranging from −7.7 nm to 7.0 nm) of myofibrils from fresh shrimp, which confirmed the remaining myofibrils with highly ordered structures in MP. The finding was consistent with myofibrils from fresh tilapia fillets [25]. For the fresh water-treated shrimp muscle tissue (Figure 3B,b), the integrated structure of myofibrils degraded into smaller filament fragments, and the aggregation and cross-linking of myofibrils were observed after frozen storage. Compared with the fresh water group, sodium tripolyphosphate (Figure 3C,c), trehalose (Figure 3D,d), and phosphorylated trehalose (Figure 3E,e) significantly enlarged the myofibrils, and fewer small fragments were found in these treatment groups, especially for the trehalose and phosphorylated trehalose groups. 

MP in shrimp muscle is susceptible to oxidation during frozen storage, producing noncovalent and/or covalent intermolecular bonds-links and leading to aggregation of MP [26]. The differences in AFM results between four treated samples after 12 weeks of frozen storage were probably caused by the different degrees of MP oxidation. Based on the results of the current study and our previous report, trehalose can inhibit the separation of the muscle bundles and maintain a relatively tight structure, thus preserving the high functionality and stability of muscle protein response to freezing and frozen storage [27]. As a potent antioxidant, the stabilization behavior of trehalose can be explained by vitrification (glass formation) theory, water replacement theory, and preferential exclusion theory [20,28]. Overall, cryoprotective (anti-oxidation) trehalose and phosphorylated trehalose preserved the integrity of the myofibrils, which were demonstrated using AFM. 

### 3.5. Hematoxylin and Eosin (H&E) Staining Analysis

**Figure 4:**
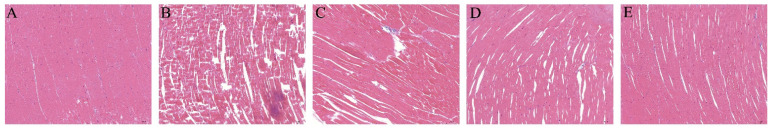
H&E staining images of transverse shrimp muscle tissue from the second abdominal segment treated with fresh water, sodium tripolyphosphate, trehalose, and phosphorylated trehalose after 12 weeks of frozen storage. (**A**): fresh shrimp muscle tissue (0 d); (**B**): shrimp muscle tissue treated with fresh water; (**C**): shrimp muscle tissue treated with sodium tripolyphosphate; (**D**): shrimp muscle tissue treated with trehalose; (**E**): shrimp muscle tissue treated with phosphorylated trehalose.

The microstructures of transverse shrimp muscle tissue from the second abdominal segment soaked in fresh water, sodium tripolyphosphate, trehalose, and phosphorylated trehalose are presented in Figure 4. The myofibrils of fresh samples (Figure 4A) were closely connected with very little space between them. However, the fresh water-treated shrimp tissue (Figure 4B) exhibited a noncoherent structure of the myofibrils with enlarged extracellular space after 12 weeks of frozen storage, revealing the weakening of mechanical strength of muscle connective tissue. This result may be explained by the physical damage caused by the growth of large ice crystals during frozen storage, leading to the degeneration of MP [29]. By comparison, the shrimp muscle fibers treated with sodium tripolyphosphate, trehalose, and phosphorylated trehalose (Figure 4C,D,F) were more tightly arranged, and the extracellular space was observably smaller than the fresh water batch. Among them, the microstructure of muscle fibers was best maintained by phosphorylated trehalose. 

During cryopreservation, ice crystals form faster outside the cell than inside, increasing in extracellular solution concentration. Myofibrils dehydrate due to counter-diffusion of solutes, downregulating the intracellular freezing point and expanding the extracellular ice crystals, thus increasing the extracellular space [30]. Consistent with the results of surface hydrophobicity, H&E staining analysis demonstrated that trehalose and phosphorylated trehalose substituted water molecules and helped maintain the native structure of shrimp muscle protein [20]. In the current study, the results of H&E staining indicated that immersing in phosphorylated trehalose had an excellent inhibition effect on the ice-crystal formation and structural preservation of shrimp muscle tissue.

### 3.6. Thermal Stability Analysis

The thermal properties of MP are correlated with the structural unfolding or aggregation of molecules that cause denaturation during freezing or frozen storage. Changes in denaturation temperature (*T_max_*) and enthalpy (*∆H*) of myosin and actin in shrimp muscle were measured during frozen storage (Table 2). 

The initial *T_max_* and Δ*H* of myosin and actin in fresh water samples at week 0 were 50.05 °C and 0.582 J/g, and 78.76 °C and 0.312 J/g, respectively, representing the maximum denaturation temperature and enthalpy of myosin head and actin. The values decreased dramatically to 44.21 °C and 0.413 J/g, and 71.15 °C and 0.240 J/g, respectively, after 12 weeks of frozen storage, demonstrating worse integrity and thermal stability of MP in shrimp in response to frozen storage [31]. 

The overall changes in sodium tripolyphosphate, trehalose, and phosphorylated trehalose batches presented similar trends (decrease) during 12 weeks storage period and exhibited better maintenance of the thermal stability of myosin and actin than fresh water. Notably, shrimp pre-soaked with phosphorylated trehalose displayed significantly higher values than those treated with sodium tripolyphosphate and trehalose at the end of storage. The two-way ANOVA (Tables S1 and S7) indicated that the *T_max_* and Δ*H* of myosin, *T_max_* of actin were significantly influenced by the cryopreservative treatment, storage time and their interaction. However, the Δ*H* of actin was only significantly influenced by the cryopreservative treatment.

A possible explanation for the results is that the interactions of saccharide and/or polyols constituents with MP (including myosin and actin) shorten exposure of buried hydrophobic residues on the protein surface, leading to improved conformational stability and structural integrity of protein [23]. Modified ice morphology influences aggregations of muscle structures which subsequently affects water loss and quality changes [32]. Adding saccharide also can decrease amount of frozen water and ice contents which reduces the negative impact of the ice on protein structural changes [33]. In addition to the antioxidant effect, phosphates can also increase the negative charges on the molecular surface by modifying several amino-acid residues on protein, resulting in the stabilization of muscle protein [34]. These findings indicated that phosphorylated trehalose has a stronger ability to maintain the thermostability properties of MP during frozen storage compared to sodium tripolyphosphate and trehalose.

## 4. Conclusions

The cryoprotective effects of phosphorylated trehalose on the quality of peeled shrimp (*Litopenaeus vannamei*) were investigated during 12 weeks of frozen storage. Compared with fresh water treated samples, treating samples with sodium tripolyphosphate, trehalose, and phosphorylated trehalose had a protective effect on MP. This includes maintaining higher WHC, MP solubility, Ca^2+^-ATPase activity and T-SH content, and reduced surface hydrophobicity. Further, phosphorylated trehalose efficiently reduced the damage to the MP structure and improved the thermal stability of MPs during frozen storage. The cryoprotection and antioxidant effects of phosphorylated trehalose on the stability of MP in frozen peeled shrimp could be used to extend the shelf life and maintain the quality of shrimp.

## Figures and Tables

**Table 1:** Effects of fresh water, sodium tripolyphosphate, trehalose, and phosphorylated trehalose on the thawing loss and cooking loss of peeled shrimp during frozen storage.

WHC	Storage Time (Week)	Fresh Water	Sodium Tripolyphosphate	Trehalose	Phosphorylated Trehalose
Thawing loss (%)	0	5.69 ± 0.21 ^Ab^	4.79 ± 0.18 ^Aa^	4.92 ± 0.20 ^Aa^	4.86 ± 0.17 ^Aa^
	3	6.23 ± 0.19 ^Bb^	4.93 ± 0.16 ^Aa^	5.01 ± 0.13 ^Aa^	4.90 ± 0.19 ^Aa^
	6	6.79 ± 0.18 ^Cc^	5.72 ± 0.14 ^Bb^	5.40 ± 0.16 ^Bab^	5.32 ± 0.11 ^Ba^
	9	7.34 ± 0.21 ^Dc^	6.12 ± 0.19 ^Cb^	5.66 ± 0.20 ^Ba^	5.44 ± 0.17 ^BCa^
	12	8.52 ± 0.20 ^Ed^	6.55 ± 0.17 ^Dc^	5.92 ± 0.15 ^Cb^	5.59 ± 0.16 ^Ca^
Cooking loss (%)	0	8.65 ± 0.24 ^Ab^	5.34 ± 0.19 ^Aa^	5.43 ± 0.21 ^Aa^	5.19 ± 0.25 ^Aa^
	3	9.53 ± 0.26 ^Bb^	5.53 ± 0.21 ^Aa^	5.61 ± 0.25 ^Aa^	5.33 ± 0.23 ^Aa^
	6	10.44 ± 0.36 ^Cc^	6.24 ± 0.23 ^Bab^	6.35 ± 0.26 ^Bb^	5.77 ± 0.19 ^Ba^
	9	11.89 ± 0.41 ^Dc^	6.89 ± 0.20 ^Cab^	7.11 ± 0.19 ^Cb^	6.50 ± 0.21 ^Ca^
	12	13.25 ± 0.37 ^Ec^	7.46 ± 0.19 ^Db^	7.54 ± 0.29 ^Cb^	6.97 ± 0.17 ^Da^

Data represent the means ± S.D. of measurement for three replicates. Duncan’s test was used to determine the significance, and the means with different upper-case (column) letters and lower--case (row) letters were significantly different at *p* < 0.05, respectively.

**Table 2:** Changes in thermal stability (*T_max_* and Δ*H*) of MP (myosin and actin) in peeled shrimp soaked with fresh water, sodium tripolyphosphate, trehalose, and phosphorylated trehalose during frozen storage.

MPs	Storage Time (Week)	Fresh Water		Sodium Tripolyphosphate		Trehalose		Phosphorylated Trehalose	
		*T_max_* (°C)	Δ*H* (J/g)	*T_max_* (°C)	Δ*H* (J/g)	*T_max_* (°C)	Δ*H* (J/g)	*T_max_* (°C)	Δ*H* (J/g)
Myosin	0	50.05 ± 0.21 ^Ea^	0.582 ± 0.012 ^Ea^	50.61 ± 0.24 ^Da^	0.600 ± 0.010 ^Ea^	50.49 ± 0.19 ^Da^	0.590 ± 0.011 ^Da^	50.66 ± 0.27 ^Da^	0.602 ± 0.009 ^Da^
	3	49.38 ± 0.20 ^Da^	0.534 ± 0.010 ^Da^	50.17 ± 0.19 ^Db^	0.576 ± 0.008 ^Db^	50.11 ± 0.28 ^Db^	0.579 ± 0.009 ^Db^	50.23 ± 0.21 ^Db^	0.586 ± 0.010 ^Db^
	6	48.11 ± 0.18 ^Ca^	0.491 ± 0.012 ^Ca^	49.26 ± 0.22 ^Cb^	0.528 ± 0.009 ^Cb^	49.10 ± 0.25 ^Cb^	0.529 ± 0.010 ^Cb^	49.38 ± 0.14 ^Cb^	0.536 ± 0.007 ^Cb^
	9	46.56 ± 0.23 ^Ba^	0.450 ± 0.011 ^Ba^	48.04 ± 0.23 ^Bb^	0.490 ± 0.011 ^Bb^	48.16 ± 0.18 ^Bbc^	0.499 ± 0.008 ^Bb^	48.60 ± 0.18 ^Bc^	0.512 ± 0.005 ^Bc^
	12	44.21 ± 0.29 ^Aa^	0.413 ± 0.012 ^Aa^	46.11 ± 0.26 ^Ab^	0.455 ± 0.010 ^Ab^	46.08 ± 0.27 ^Ab^	0.458 ± 0.011 ^Ab^	47.22 ± 0.23 ^Ac^	0.478 ± 0.007 ^Ac^
Actin	0	78.76 ± 0.31 ^Ea^	0.312 ± 0.004 ^Da^	79.01 ± 0.32 ^Dab^	0.315 ± 0.003 ^Da^	78.89 ± 0.26 ^Da^	0.318 ± 0.005 ^Da^	79.38 ± 0.30 ^Db^	0.322 ± 0.003 ^Da^
	3	77.21 ± 0.22 ^Da^	0.308 ± 0.005 ^Da^	78.90 ± 0.24 ^Db^	0.311 ± 0.004 ^Da^	78.62 ± 0.31 ^Db^	0.310 ± 0.003 ^Da^	78.99 ± 0.22 ^Db^	0.313 ± 0.002 ^Ca^
	6	76.06 ± 0.27 ^Ca^	0.286 ± 0.004 ^Ca^	77.67 ± 0.36 ^Cb^	0.298 ± 0.002 ^Cb^	77.88 ± 0.29 ^Cb^	0.301 ± 0.003 ^Cb^	77.70 ± 0.34 ^Cb^	0.300 ± 0.004 ^Bb^
	9	74.36 ± 0.35 ^Ba^	0.264 ± 0.003 ^Ba^	75.83 ± 0.27 ^Bb^	0.280 ± 0.004 ^Bb^	76.65 ± 0.34 ^Bc^	0.290 ± 0.002 ^Bc^	76.77 ± 0.28 ^Bc^	0.292 ± 0.003 ^Bc^
	12	71.15 ± 0.28 ^Aa^	0.240 ± 0.005 ^Aa^	74.53 ± 0.24 ^Ab^	0.263 ± 0.003 ^Ab^	75.09 ± 0.21 ^Ac^	0.271 ± 0.004 ^Ab^	75.41 ± 0.20 ^Ad^	0.281 ± 0.002 ^Ac^

Mean ± standard deviation of triplicate determinations (*n* = 3). Values with different capital letters in the same column denote significant differences (*p* < 0.05) as a result of the frozen storage time; different lowercase letters in the same row for *T_max_* and Δ*H* denote significant differences (*p* < 0.05) as a result of previous soaking treatment.

## Data Availability

Data are contained within this article.

## Supplementary Materials

The following supporting information can be downloaded at: https://www.mdpi.com/article/10.3390/foods11203189/s1, Table S1. P values of two-way ANOVA for main effects and interactions for the different variables measured in peeled shrimp.; Table S2. Two-way ANOVA for WHC including thawing loss and cooking loss of peeled shrimp.; Table S3. Two-way ANOVA for myofibrillar proteins (MP) content in peeled shrimp.; Table S4. Two-way ANOVA for Ca^2+^-ATPase activity of MP in peeled shrimp. Table S5. Two-way ANOVA for total sulphydryl (T-SH) content of MP in peeled shrimp. Table S6. Two-way ANOVA for surface hydrophobicity of MP in peeled shrimp. Table S7. Two-way ANOVA for the thermal stability (*Tmax* and Δ*H*) of myosin and actin in peeled shrimp.

## References

[1] Zhang B., Yao H., Qi H., Ying X.G. (2020). Cryoprotective characteristics of different sugar alcohols on peeled Pacific white shrimp (*Litopenaeus vannamei*) during frozen storage and their possible mechanisms of action. Int. J. Food Prop..

[2] Oliveira M.E.d.S., Gonçalves A.A. (2019). The effect of different food grade additives on the quality of Pacific white shrimp (*Litopenaeus vannamei*) after two freeze-thaw cycles. LWT.

[3] Thangavelu K.P., Kerry J.P., Tiwari B.K., McDonnell C.K. (2019). Novel processing technologies and ingredient strategies for the reduction of phosphate additives in processed meat. Trends Food Sci. Technol..

[4] Jain N.K., Roy I. (2009). Effect of trehalose on protein structure. Protein Sci..

[5] Zhang B., Yao H., Qi H., Zhang X.L. (2020). Trehalose and alginate oligosaccharides increase the stability of muscle proteins in frozen shrimp (*Litopenaeus vannamei*). Food Funct..

[6] Xu Y., Wu Y.J., Sun P.L., Zhang F.M., Linhardt R.J., Zhang A.Q. (2019). Chemically modified polysaccharides: Synthesis, characterization, structure activity relationships of action. Int. J. Biol. Macromol..

[7] Zhang M., Su N., Huang Q., Zhang Q., Wang Y., Li J., Ye M. (2017). Phosphorylation and antiaging activity of polysaccharide from *Trichosanthes* peel. J. Food Drug Anal..

[8] Cao Y.Y., Ji Y.H., Liao A.M., Huang J.H., Thakur K., Li X.L., Hu F., Zhang J.G., Wei Z.J. (2020). Effects of sulfated, phosphorylated and carboxymethylated modifications on the antioxidant activities in-vitro of polysaccharides sequentially extracted from Amana *edulis*. Int. J. Biol. Macromol..

[9] Liu Z., Xu D., Xia N., Zhao X., Kong F., Wang S., Fatehi P. (2018). Preparation and application of phosphorylated xylan as a flocculant for cationic ethyl violet dye. Polymers.

[10] Teng Q., Hu X.F., Luo F., Cheng C., Ge X.Y., Yang M.Y., Liu L.M. (2016). Influences of introducing frogs in the paddy fields on soil properties and rice growth. J. Soils Sediments.

[11] Zhang B., Wu H.X., Yang H.C., Xiang X.W., Li H.B., Deng S.G. (2017). Cryoprotective roles of trehalose and alginate oligosaccharides during frozen storage of peeled shrimp (*Litopenaeus vannamei*). Food Chem..

[12] Zhang B., Yang H., Tang H., Hao G., Zhang Y., Deng S. (2017). Insights into cryoprotective roles of carrageenan oligosaccharides in peeled whiteleg shrimp (*Litopenaeus vannamei*) during frozen storage. J. Agric. Food Chem..

[13] Ying X.G., Wu Q.J., Shui S.S., Zhang B., Benjakul S. (2021). Insights into the similarities and differences of whiteleg shrimp pre-soaked with sodium tripolyphosphate and sodium trimetaphosphate during frozen storage. Food Chem..

[14] Zuo H., Han L., Yu Q., Niu K., Zhao S., Shi H. (2016). Proteome changes on water-holding capacity of yak *longissimus lumborum* during postmortem aging. Meat Sci..

[15] Li B., Sun D.W. (2002). Novel methods for rapid freezing and thawing of foods—A review. J. Food Eng..

[16] Reza M.S., Bapary M.A., Ahasan C.T., Islam M.N., Kamal M. (2009). Shelf life of several marine fish species of Bangladesh during ice storage. Int. J. Food Sci. Technol..

[17] Shui S.S., Qi H., Shaimaa H., Aubourg S.P., Zhang B. (2021). Kappa-carrageenan and its oligosaccharides maintain the physicochemical properties of myofibrillar proteins in shrimp mud (Xia-Hua) during frozen storage. J. Food Sci..

[18] Li S., Xiong Q., Lai X., Li X., Wan M., Zhang J., Yan Y., Cao M., Lu L., Guan J. (2016). Molecular modification of polysaccharides and resulting bioactivities. Compr. Rev. Food Sci. Food Saf..

[19] Gao W., Hou R., Zeng X.A. (2018). Synergistic effects of ultrasound and soluble soybean polysaccharide on frozen surimi from grass carp. J. Food Eng..

[20] Jain N.K., Roy I. (2010). Trehalose and protein stability. Curr. Protoc. Protein Sci..

[21] Shao Y., Wang L., Chen C., Xiong G., Hu Y., Qiao Y., Wu W., Li X., Wang J., Liao L. (2018). Antioxidant capacity of fermented soybeans and their protective effect on protein oxidation in largemouth bass (*Micropterus salmoides*) during repeated freezing-thawing (FT) treatments. LWT-Food Sci. Technol..

[22] Lu H., Zhang L., Li Q., Luo Y. (2017). Comparison of gel properties and biochemical characteristics of myofibrillar protein from bighead carp (*Aristichthys nobilis*) affected by frozen storage and a hydroxyl radical-generation oxidizing system. Food Chem..

[23] Nikoo M., Benjakul S., Rahmanifarah K. (2016). Hydrolysates from marine sources as cryoprotective substances in seafoods and seafood products. Trends Food Sci. Technol..

[24] Zhang B., Fang C., Hao G., Zhang Y. (2018). Effect of kappa-carrageenan oligosaccharides on myofibrillar protein oxidation in peeled shrimp (*Litopenaeus vannamei*) during long-term frozen storage. Food Chem..

[25] Zhao X., Zhou Y., Zhao L., Chen L., He Y., Yang H. (2019). Vacuum impregnation of fish gelatin combined with grape seed extract inhibits protein oxidation and degradation of chilled tilapia fillets. Food Chem..

[26] Nikoo M., Benjakul S. (2015). Potential application of seafood-derived peptides as bifunctional ingredients, antioxidant-cryoprotectant: A review. J. Funct. Foods.

[27] Zhang B., Zhao J.L., Chen S.J., Zhang X.L., Wei W.Y. (2019). Influence of trehalose and alginate oligosaccharides on ice crystal growth and recrystallization in whiteleg shrimp (*Litopenaeus vannamei*) during frozen storage with temperature fluctuations. Int. J. Refrig..

[28] Zhang B., Qi X.E., Mao J.L., Ying X.G. (2020). Trehalose and alginate oligosaccharides affect the stability of myosin in whiteleg shrimp (*Litopenaeus vannamei*): The water-replacement mechanism confirmed by molecular dynamic simulation. LWT-Food Sci. Technol..

[29] Zhang B., Cao H.J., Lin H.M., Deng S.G., Wu H. (2019). Insights into ice-growth inhibition by trehalose and alginate oligosaccharides in peeled Pacific white shrimp (*Litopenaeus vannamei*) during frozen storage. Food Chem..

[30] Ma L.k., Zhang B., Deng S.g., Xie C. (2015). Comparison of the cryoprotective effects of trehalose, alginate, and its oligosaccharides on peeled shrimp (*Litopenaeus vannamei*) during frozen storage. J. Food Sci..

[31] Wang B., Kong B., Li F., Liu Q., Zhang H., Xia X. (2020). Changes in the thermal stability and structure of protein from porcine longissimus dorsi induced by different thawing methods. Food Chem..

[32] Harnkarnsujarit N., Charoenrein S. (2011). Influence of collapsed structure on stability of β-carotene in freeze-dried mangoes. Food Res. Int..

[33] Harnkarnsujarit N., Kawai K., Suzuki T. (2015). Effects of freezing temperature and water activity on microstructure, color, and protein conformation of freeze-dried bluefin tuna (*Thunnus orientalis*). Food Bioprocess Technol..

[34] Hu Z., Qiu L., Sun Y., Xiong H., Ogra Y. (2019). Improvement of the solubility and emulsifying properties of rice bran protein by phosphorylation with sodium trimetaphosphate. Food Hydrocoll..

