# Supplementary material for: Phosphorylated Trehalose Suppresses the Denaturation of Myofibrillar Proteins in Peeled Shrimp (Litopenaeus vannamei) during Long-Term Frozen Storage
Source: Foods. 2022 Oct 13;11(20):3189. doi: 10.3390/foods11203189 (PMC9602034; doi:10.3390/foods11203189)
Supplement: Supplementary file 1 [file foods-11-03189-s001.zip › foods-1924806-supplementary.pdf]

Table S1 P values of Two-way ANOVA for main effects and interactions for the different variables measured in peeled shrimp.

**Source of Variation**	**WHC (Thawing loss)**	**WHC (Cooking loss)**	**Myofibrillar proteins content**	**Ca^{2+}-ATPase activity**	**Total sulphydryl content**	**Surface hydrophobicity**	**Thermal stability (Myosin T_{max})**	**Thermal stability (Myosin ΔH)**	**Thermal stability (Actin T_{max})**	**Thermal stability (Actin ΔH)**
Cryopreservative treatment	3.495E-24	6.759E-27	1.700E-25	1.435E-31	4.894E-28	0.0375	1.532E-37	3.173E-32	1.669E-33	0.0375
Storage time	2.962E-27	3.011E-39	2.621E-17	1.602E-18	4.451E-13	0.2539	5.792E-21	5.022E-16	1.057E-18	0.2539
Interaction	3.168E-10	1.121E-09	2.548E-09	6.596E-08	0.0002689	0.4252	1.476E-08	0.02491	1.759E-08	0.4252

Table S2 Two-way ANOVA for WHC including thawing loss and cooking loss of peeled shrimp.

**1.Two-way ANOVA results (Thawing loss)**								
1	Table Analyzed	**Thawing loss Data**						
2								
3	Two-way ANOVA	Ordinary						
4	Alpha	0.05						

5							
6	Source of Variation	SS	DF	MS	F	P value	F crit
7	Cryopreservative treatment	19.88	4	4.970	162.9	3.495E-24	2.606
8	Storage time	26.84	3	8.946	293.2	2.962E-27	2.839
9	Interaction	4.725	12	0.3937	12.90	3.168E-10	2.003
10	Residual	1.221	40	0.03051			
11							
12	Data summary						
13	Number of columns (Cryopreservative treatment)	4					
14	Number of rows (Storage time)	5					
15	Number of values	60					
**2.Two-way ANOVA results (Cooking loss)**							
1	Table Analyzed	**Cooking loss Data**					
2							
3	Two-way ANOVA	Ordinary					
4	Alpha	0.05					
5							

6	Source of Variation	SS	DF	MS	F	P value	F crit
7	Cryopreservative treatment	58.29	4	14.57	226.4	6.759E-27	2.606
8	Storage time	233.0	3	77.67	1206	3.011E-39	2.839
9	Interaction	9.147	12	0.7622	11.84	1.121E-09	2.003
10	Residual	2.574	40	0.06435			
11							
12	Data summary						
13	Number of columns (Cryopreservative treatment)	4					
14	Number of rows (Storage time)	5					
15	Number of values	60					

Table S3 Two-way ANOVA for myofibrillar proteins (MP) content in peeled shrimp.

**1.Two-way ANOVA results (MP content)**							
1	Table Analyzed	**MP content Data**					
2							
3	Two-way ANOVA	Ordinary					
4	Alpha	0.05					
5							

6	Source of Variation	SS	DF	MS	F	P value	F crit
7	Cryopreservative treatment	7276	4	1819	191.2	1.700E-25	2.606
8	Storage time	2397	3	799.0	83.95	2.621E-17	2.839
9	Interaction	1278	12	106.5	11.19	2.548E-09	2.003
10	Residual	380.7	40	9.517			
11							
12	Data summary						
13	Number of columns (Cryopreservative treatment)	4					
14	Number of rows (Storage time)	5					
15	Number of values	60					

Table S4 Two-way ANOVA for Ca^{2+}-ATPase activity of MP in peeled shrimp.

**1.Two-way ANOVA results (Ca^{2+}-ATPase activity)**							
1	Table Analyzed	**Ca^{2+}-ATPase activity Data**					
2							
3	Two-way ANOVA	Ordinary					

4	Alpha	0.05					
5							
6	Source of Variation	SS	DF	MS	F	P value	F crit
7	Cryopreservative treatment	0.05784	4	0.01446	395.3	1.435E-31	2.606
8	Storage time	0.01082	3	0.0036	98.60	1.602E-18	2.839
9	Interaction	0.003881	12	0.0003	8.841	6.596E-08	2.003
10	Residual	0.001463	40	3.658E-05			
11							
12	Data summary						
13	Number of columns (Cryopreservative treatment)	4					
14	Number of rows (Storage time)	5					
15	Number of values	60					

Table S5 Two-way ANOVA for total sulphydryl (T-SH) content of MP in peeled shrimp.

**1.Two-way ANOVA results (T-SH content)**							
1	Table Analyzed	**T-SH content Data**					

2							
3	Two-way ANOVA	Ordinary					
4	Alpha	0.05					
5							
6	Source of Variation	SS	DF	MS	F	P value	F crit
7	Cryopreservative treatment	1083	4	270.8	259.7	4.894E-28	2.606
8	Storage time	144.8	3	48.28	46.30	4.451E-13	2.839
9	Interaction	52.97	12	4.414	4.233	0.0002689	2.003
10	Residual	41.71	40	1.043			
11							
12	Data summary						
13	Number of columns (Cryopreservative treatment)	4					
14	Number of rows (Storage time)	5					
15	Number of values	60					

Table S6 Two-way ANOVA for surface hydrophobicity of MP in peeled shrimp.

**1.Two-way ANOVA results (Surface hydrophobicity)**						

1	Table Analyzed	**Surface hydrophobicity Data**					
2							
3	Two-way ANOVA	Ordinary					
4	Alpha	0.05					
5							
6	Source of Variation	SS	DF	MS	F	P value	F crit
7	Cryopreservative treatment	9015	4	2539	10.09	0.0375	2.606
8	Storage time	986.3	3	328.9	1.411	0.2539	2.839
9	Interaction	2938	12	244.9	1.051	0.4252	2.003
10	Residual	9325	40	233.1			
11							
12	Data summary						
13	Number of columns (Cryopreservative treatment)	4					
14	Number of rows (Storage time)	5					
15	Number of values	60					

Table S7 Two-way ANOVA for the thermal stability (T_{max} and ΔH) of myosin and actin in peeled shrimp

**1.Two-way ANOVA results (Myosin T_{max})**							
1	Table Analyzed	**Myosin T_{max} Data**					
2							
3	Two-way ANOVA	Ordinary					
4	Alpha	0.05					
5							
6	Source of Variation	SS	DF	MS	F	P value	F crit
7	Cryopreservative treatment	159.7	4	39.91	796.5	1.532E-37	2.606
8	Storage time	20.30	3	6.768	135.0	5.792E-21	2.839
9	Interaction	5.939	12	0.4949	9.876	1.476E-08	2.003
10	Residual	2.005	40	0.0501			
11							
12	Data summary						
13	Number of columns (Cryopreservative treatment)	4					
14	Number of rows (Storage time)	5					
15	Number of values	60					

**2.Two-way ANOVA results (Myosin ΔH)**							
1	Table Analyzed	**Myosin ΔH Data**					
2							
3	Two-way ANOVA	Ordinary					
4	Alpha	0.05					
5							
6	Source of Variation	SS	DF	MS	F	P value	F crit
7	Cryopreservative treatment	0.1617	4	0.04042	427.1	3.173E-32	2.606
8	Storage time	0.02003	3	0.006676	70.55	5.022E-16	2.839
9	Interaction	0.002600	12	0.0002167	2.290	0.02491	2.003
10	Residual	0.003785	40	9.463E-05			
11							
12	Data summary						
13	Number of columns (Cryopreservative treatment)	4					
14	Number of rows (Storage time)	5					
15	Number of values	60					
**3.Two-way ANOVA results (Actin T_{max})**							

1	Table Analyzed	**Actin T_{max} Data**					
2							
3	Two-way ANOVA	Ordinary					
4	Alpha	0.05					
5							
6	Source of Variation	SS	DF	MS	F	P value	F crit
7	Cryopreservative treatment	0.02377	4	0.005941	496.5	1.669E-33	2.606
8	Storage time	0.003624	3	0.001208	101.0	1.057E-18	2.839
9	Interaction	0.001400	12	0.0001167	9.7510	1.759E-08	2.003
10	Residual	0.0004787	40	1.197E-05			
11							
12	Data summary						
13	Number of columns (Cryopreservative treatment)	4					
14	Number of rows (Storage time)	5					
15	Number of values	60					
**4.Two-way ANOVA results (Actin ΔH)**							
1	Table Analyzed	**Actin ΔH Data**					
2							

3	Two-way ANOVA	Ordinary					
4	Alpha	0.05					
5							
6	Source of Variation	SS	DF	MS	F	P value	F crit
7	Cryopreservative treatment	9015	4	2539	10.09	0.0375	2.606
8	Storage time	986.3	3	328.9	1.411	0.2539	2.839
9	Interaction	2938	12	244.9	1.051	0.4252	2.003
10	Residual	9325	40	233.1			
11							
12	Data summary						
13	Number of columns (Cryopreservative treatment)	4					
14	Number of rows (Storage time)	5					
15	Number of values	60					
